# Molecular Screening of Microorganisms Associated with Discolored Wood in Dead European Beech Trees Suffered from Extreme Drought Event Using Next Generation Sequencing

**DOI:** 10.3390/plants10102092

**Published:** 2021-10-02

**Authors:** Witoon Purahong, Benjawan Tanunchai, Sara Fareed Mohamed Wahdan, François Buscot, Ernst-Detlef Schulze

**Affiliations:** 1 UFZ-Helmholtz Centre for Environmental Research, Department of Soil Ecology, Theodor-Lieser-Str. 4, D-06120 Halle, Germany; tanunchai.benjawan@ufz.de (B.T.); sara-fareed-mohamed.wahdan@ufz.de (S.F.M.W.); francois.buscot@ufz.de (F.B.); 2 Botany Department, Faculty of Science, Suez Canal University, Ismailia 41522, Egypt; 3 German Centre for Integrative Biodiversity Research (iDiv) Halle-Jena-Leipzig, Deutscher Platz 5e, D-04103 Leipzig, Germany; 4 Max Planck Institute for Biogeochemistry, Hans-Knöll-Str. 10, D-07745 Jena, Germany; dschulze@bgc-jena.mpg.de

**Keywords:** climate change, *Fagus sylvatica*, Illumina sequencing, meta-barcoding, plant pathogenic bacteria, plant pathogenic fungi

## Abstract

Drought events weaken trees and make them vulnerable to attacks by diverse plant pathogens. Here, we propose a molecular method for fast screening of microorganisms associated with European beech decline after an extreme drought period (2018) in a forest of Thuringia, Germany. We used Illumina sequencing with a recent bioinformatics approach based on DADA2 to identify archaeal, bacterial, and fungal ASVs (amplicon sequence variants) based on bacterial and archaeal 16S and fungal ITS genes. We show that symptomatic beech trees are associated with both bacterial and fungal plant pathogens. Although the plant pathogen sequences were detected in both discolored and non-discolored wood areas, they were highly enriched in the discolored wood areas. We show that almost each individual tree was associated with a different combination of pathogens. *Cytospora* spp. and *Neonectria coccinea* were among the most frequently detected fungal pathogens, whereas *Erwinia* spp. and *Pseudomonas* spp. were the dominant bacterial plant pathogens. We demonstrate that bacterial plant pathogens may be of major importance in beech decline.

## 1. Introduction

Drought is a complex multivariate environmental phenomenon that is presently intensified by climate changes [1,2]. Drought events are predicted to occur with higher intensity, duration, and frequency in the future, with expected negative impacts on plant health and productivity in both agricultural and forest ecosystems [1,2,3]. Forest ecosystems especially are more sensitive to drought as compared with agricultural land, as forest trees are long-lived with harvest after a century, and irrigation is generally impossible and not economically practical [4]. The drought event in 2003 was referred to the most severe drought in Europe for the last 500 years [5]; however, the 2018 drought event that occurred in many European countries (including Austria, Germany and Switzerland) broke this record in duration and intensity [5]. The mean air temperature from April to October in 2018 exceeded the long-term average by 3.3 °C, and was approximately 1.2 °C higher than in 2003. The extreme drought in 2018 had a much higher negative impact on forest ecosystems than the one of 2003 [5]. The drought event in 2018 not only caused immediate large-scale mortality of many tree species including both broadleaved (*Fagus sylvatica*) and coniferous (*Picea abies*) trees, but also had drought-legacy effects on tree health in the subsequent year, 2019 [5]. During the drought period in 2019 (a summer after extreme drought period in 2018), many European beech trees were killed by an unknown plant pathogen-complex, which contributed to high economic and ecological losses [5]. It has been hypothesized that the 2018 drought event weaken the trees making them vulnerable to insect pests and fungal pathogens [5]. Although it is clear that tree diseases have been detected more often after 2018, the related disease agents have not been fully assessed. Fungi may not be the only disease agents, as the severity and progression of bacterial diseases are also found to be enhanced by drought [6]. The processes to identify the correct pathogens causing specific diseases on each tree species require time, from sample collection, microbial isolation, purification, characterization and re-inoculation to fulfilling Koch’s postulates. These processes are needed, however they rely also on culturing techniques, which are known to capture only a small part of microbial communities [7]. Some plant pathogens, even dominant ones, can be overlooked depending on the cultural media and isolation efforts [7]. Therefore, a full assessment of the species assembly living in trees exposed to pathogenic attacks becomes an essential first step in such analyses.

Next-generation sequencing (NGS) technologies have made great progress in detecting and identifying plant pathogenic microbes, as well as other microbial functional groups directly in various environmental sample types, including soils, roots, wood, and leaves [7,8,9,10]. The accuracy of microbial identification is generally accepted at the genus level [9,11]; however, depending on primers, bioinformatics and completeness of the database used, species identification from NGS is also possible [12]. Although results from NGS alone cannot be used to fulfil Koch’s postulates, it does provide fast and accurate information on which microbes are present and which could potentially cause diseases [13]. Furthermore, in nature, different pathogens co-occur and interact with each other and the pathogenicity of a specific pathogen may depend on their abundances, as well as the abundances of other co-occurring pathogens [14,15]. NGS enables to obtain the data of complex microbial communities, including all potential plant pathogens, independent from culturing approaches [7,10,14]. Thus, NGS would be a prime technology to identify the status quo of an infection. Nevertheless, biases introduced by primer selection and PCR can be considered as important shortcomings of NGS [16]. For examples, universal ITS primer sets for fungi may have limited ability to identify plant pathogens of different taxa, especially oomycetes [17]. Such flaws can be partly overcome by using specific primers for the known target taxa, which may not be amplified by such universal primers. Furthermore, due to PCR biases, not all microorganisms are captured by NGS with the same efficiency. Overall, the results obtained by NGS have to be interpreted carefully. The addition of other molecular approaches should confirm and validate those NGS results [18]. In any case, data can only be interpreted within the range of certainty of the PCR primers.

In this study, we aimed to screen for microorganisms associated with European beech decline after the extreme drought period of 2018 in a forest of Thuringia, Germany, using Illumina sequencing targeting the bacterial and archaeal 16S and fungal ITS genes. In 2019, beech decline disease spread to large land areas, causing damage between 20–30% of the total European beech population [19]. Here we analyzed wood samples of freshly felled of *Fagus sylvatica* L. trees. We adopted a recent bioinformatics approach based on DADA2 and identified archaeal, bacterial, and fungal ASVs (amplicon sequence variants) [20,21]. At our test site, the disease was visible as red to dark brown stains in the wood cross-section (discolored wood). The size of the stained segments was largest close to the ground and disappeared further up (no such stain at 4-m height). We hypothesized that various fungal and bacterial plant pathogens can be detected in discolored wood areas as compared with non-discolored wood areas [22]. *Phytophthora* spp. and *Neonectria coccinea* may be among the frequently detected microfungal pathogens that attack weak European beech trees that suffer from drought event [5]. Based on the symptoms observed from our study at the Thüringen forest, we hypothesized that the discolored wood is caused by *Diplodia* sp. and/or *Neonectria coccinea*, as reported before in other German forest sites [23]. Thus, our work also aimed to confirm this diagnosis.

## 2. Materials and Methods

### 2.1. Study Site and Sampling Methodology

**Figure 1:**
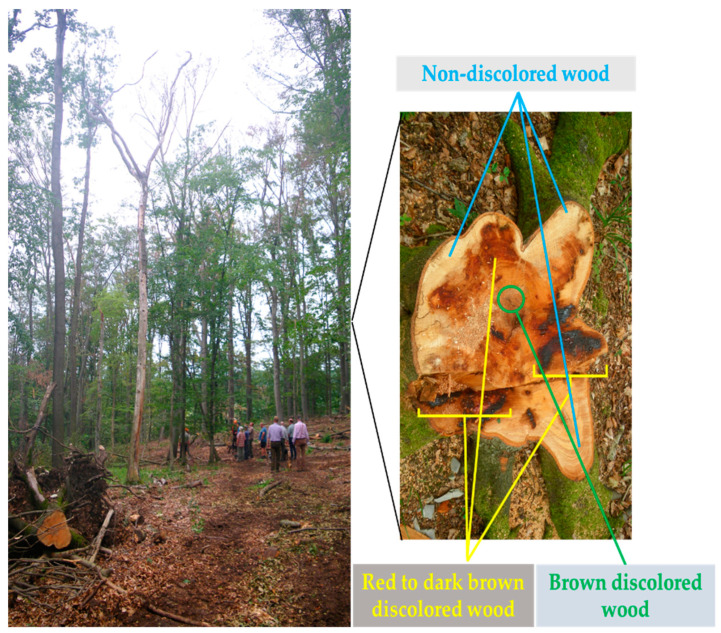
Dieback of European beech (*Fagus sylvatica* L.) trees (**left**) after extreme drought in a Thüringen forest (Schulze, 09.08.2019). Cross-section of beech wood stump (**right**) shows examples of discolored (brown discoloration area in the tree center (green) and irregular red to dark brown discoloration areas (yellow)) and non-discolored (blue) areas. Brown discoloration is known to be caused by oxygen and entering wounds [22]. The red to dark brown discoloration areas may be associated with *Neonectria coccinea* [23] and additional organisms (this study).

The experiment was conducted at a Thüringen forest (co-ordinate: 51.3797 N, 10.5274 E) (Figure 1). The studied site is located at the Dün, an east–west stretching limestone reef in Northern Thuringia. At this site (9.8 ha), the shallow soils close to the rim were predominantly affected. The forest was dominated by European beech trees (*Fagus sylvatica* L.). In this exploratory study, we chose to look at the variations in pathogens at one site, rather than looking at a regional scale. A total of 85 stems were cut in 2019 from trees that had visible signs of crown-dieback. The forest floor was dominated by regeneration of *Acer*, *Fraxinus* and *Fagus*, and by herbaceous species of a mesic limestone flora (dominance of *Allium ursinum* in early summer). In 2019, beech decline disease spread to the whole forest, causing damage to 20–30% of the total European beech population. We sampled wood stumps from 5 diseased European beech trees with different diameters, from 25 to 41 cm, when they were freshly felled. We clearly observed red to dark brown stains in wood (discolored wood) starting from the root upward to the stem. The formation of discolored areas in the woody tissues of trees is generally initiated by wound (caused by physical injury, as well as animal, insects and plant pathogens) and broken branches, which produce an open route for oxygen to penetrate to the central parts of the stem [22,24]. The discolored areas in the woody tissues of European beech are targets for microorganisms that can eventually cause decay [22].

In this study, the focus was not on the brown discoloration in the tree center, but on the red to dark brown stains, which were previously identified to be associated with *Neonectria coccinea* [23]. In the laboratory, we used a cordless drill equipped with a wood auger (diameter: 10 mm) to sample the inner bark and discolored wood areas (red to dark brown stained wood) from each of wood stump (later referred to as discolored wood samples 1–5; D1–D5) (Figure 1, Supplementary Figure S1). The wood auger was dipped for 3 min into 70% ethanol, flamed and wiped with ethanol between drillings to avoid cross-contamination [25]. For comparison, we also sampled the inner bark and non-discolored wood areas of each wood stump using a new wood auger (later referred to as non-discolored wood samples 1–5; ND1–ND5) (Figure 1, Supplementary Figure S1). For each sample, wood from 3 sampling areas (discolored wood samples: inner bark and cambium (1 subsample from 3 drills), sapwood (1 subsample from 3 drills) and heartwood (1 subsample from 3 drills); non-discolored wood samples: inner bark, cambium and/or sapwood (3 subsamples from 9 drills)) were homogenized and 150 mg of subsample was used for DNA extraction. In total, 10 homogenized composite wood samples were used for DNA extraction.

### 2.2. DNA Extraction and Molecular Detection of Microbial Communities

DNA was extracted from each homogenized composite wood sample using DNeasy PowerSoil Kit (Qiagen, Hilden, Germany) according to the manufacturer’s instructions, with the aid of a Precellys 24 tissue homogenizer (Bertin Instruments, Montigny-le-Bretonneux, France). The presence and quantity of genomic DNA was checked using a NanoDrop ND-1000 spectrophotometer (Thermo Fisher Scientific, Dreieich, Germany), and the extracts were stored at −20 °C.

Amplicon library preparation for bacteria and fungi, PCR, Illumina sequencing and bioinformatics are described in our previous publications [21,26]. Briefly, for construction of the bacterial and archaeal amplicon libraries, the 16S rRNA gene V4 region was amplified using the universal bacterial/archaeal primer pair 515F (5′-GTGCCAGCMGCCGCGGTAA-3′) and 806R (5′-GGACTACHVGGGTWTCTAAT-3′) [27] with Illumina adapter sequences. For establishing fungal amplicon libraries, the fungal ITS2 gene was amplified using the fungal primer pair fITS7 (5-GTGARTCATCGAATCTTTG-3) [28] and ITS4 primer (5-TCCTCCGCTTATTGATATGC-3) [29] with Illumina adapter sequences. Amplifications were performed using 20 µL reaction volumes with 5× HOT FIRE Pol Blend Master Mix (Solis BioDyne, Tartu, Estonia). The products from three technical replicates were then pooled in equimolar concentrations. Paired-end sequencing (2 × 300 bp) was performed on the pooled PCR products using a MiSeq Reagent kit v3 on an Illumina MiSeq system (Illumina Inc., San Diego, CA, USA) at the Department of Soil Ecology, Helmholtz Centre for Environmental Research, Germany. The raw 16S and ITS rRNA gene sequences were deposited at the National Center for Biotechnology Information (NCBI) Sequence Read Archive under the accession number PRJNA722031.

**Figure 2:**
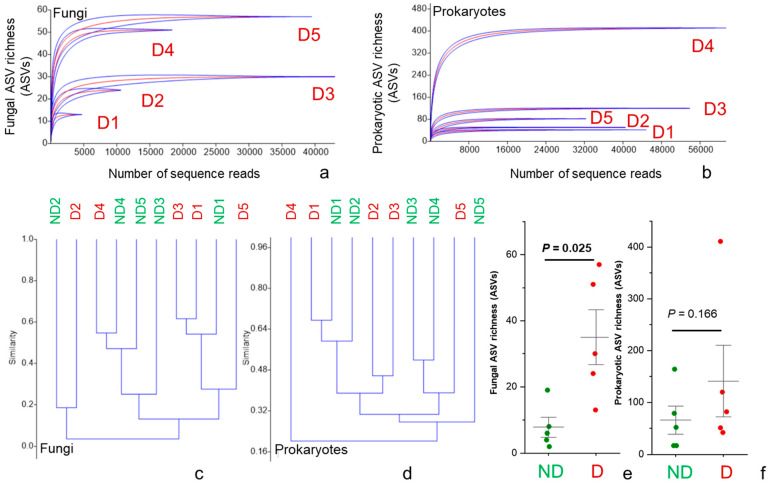
Individual sample rarefaction curves for fungi (**a**) and bacteria (**b**) cluster analysis based on Bray–Curtis similarity index for fungi (**c**) and bacteria (**d**) and ASV richness of fungi (**e**) and bacteria (**f**). ND = non-discolored wood samples (green) and D = discolored wood samples (red).

The 16S and ITS rDNA sequences corresponding to the forward and reverse primers were trimmed from the demultiplexed raw reads using cutadapt [30]. Paired-end sequences were quality-trimmed, filtered for chimeras, and merged using the DADA2 package [20] through the pipeline dadasnake [21]. Assembled reads fulfilling the following criteria were retained for further analyses: a minimum length of 100 nt (bacteria) or 70 nt (fungi), quality scores at least equal to 9 with maximum expected error score of 0.5 (bacteria) and 5 (fungi) for forward and reverse sequences and no ambiguous nucleotides. Merging was conducted with 0 or 2 mismatches allowed and a minimum overlap of 12 or 20 nucleotides required for bacterial and fungal sequences, respectively. High-quality reads were clustered into amplicon sequence variants (ASVs) for prokaryotes and fungi after chimera removal. The SILVA SSU database v. 138 was used for taxonomic classification of the bacterial and archaeal ASVs. Fungal ASVs were classified against the UNITE v7.2 database [31]. Both sets of ASVs were classified using the Bayesian classifier as implemented in the mothur classify.seqs command, with a cut-off of 60. The ASV method was used to infer the biological sequences in the sample, as described previously [32]. Rare ASVs (singletons), which potentially represent artificial sequences, were removed. Microbial taxonomic and relative abundance information are provided in Supplementary Tables S1 and S2. The rarefaction curves of all the samples reached saturation (Figure 2a,b; Supplementary Figure S2); thus, we used the observed richness as a measure of microbial diversity. We checked and confirmed the taxonomic annotations of the top 20 most abundant fungal ASVs (highest relative abundances detected in discolored areas) in this study by conducting BLAST searches against the current version of UNITE (version: 8.2; 15 January 2020), and the UNITE species hypotheses [33] were used for all 20 ASVs. The fungal ecological function of each ASV was determined using FUNGuild [34] and rechecked with publications listed in the Web of Science (ISI Web of Knowledge). We verified and confirmed the taxonomic annotations at the species level for the 20 most abundant bacterial ASVs in this study by conducting BLAST searches against the current version of NCBI (version: 2.11.0). Only the top and consistent match with 100% identity was considered.

We also used *Phytophthora* genus and species-specific primers to validate the existence/absence of *Phytophthora* spp. in wood samples [35]. Primer pair YPh1F (5′-CGACCATKGGTGTGGACTTT-3′) and YPh2R (5′-ACGTTCTCMCAGGCGTATCT-3′) was used for detecting general *Phytophthora* spp. *Phytophthora* species-specific primers were included (i) primer pair Ycam4F (5′- TGGCTAAGTTTTGACCTCCAG-3′) and Ycam3R (5′- ACAATTCCGAATAATCACAGTGTA -3′) for *Phytophthora cambivora*; (ii) primer pair Ycac1F (5′- CCATACAAAATTCTGCGCTAGG -3′) and Ycac2R (5′- AGACACACAAGTGGACCGTTAG -3′) for *Phytophthora cactorum*; and (iii) primer pair Ycit1F (5′- TCCAACTTAGTAAGAGTGCTGGA -3′) and Ycit2R (5′- CAACAGAAATCCTGAAGTACTGTATCA -3′) for *Phytophthora citricola*. All PCR conditions and protocols are described elsewhere [35].

### 2.3. Statistics

All statistical analyses were performed using PAST version 2.17 [36]. Individual sample rarefaction curves were plotted using diversity function. Cluster analysis based on Bray–Curtis distance measure was performed using the “multivar” function. Comparisons of fungal and bacterial richness detected between discolored and non-discolored wood areas were analyzed using the paired sample *t*-test. Normality and equality of group variances were determined using the Jarque–Bera test and *F* test.

## 3. Results and Discussion

We detected high numbers of fungal and prokaryotic (bacteria and archaeal) sequences in all wood samples collected from discolored areas (D samples, minimum number of sequences = 4881 and 32,406 sequences per sample for fungi and bacteria, respectively) (Figure 2). In non-discolored areas (ND samples), we also detected very high number of plant sequences with much lower fungal and prokaryotic sequences (ranging between 43–529 and 416–21,083 for fungi and prokaryotes). The rarefaction curves of fungi and prokaryotes showed saturation of both sample types (Figure 2 and Figure S2).

Discolored wood samples had a significantly higher fungal richness (*t* = −3.48, *p* = 0.025) as compared with non-discolored wood samples, whereas bacterial richness was not significantly different (*t* = 1.69, *p* = 0.166) (Figure 2e,f). Prokaryotic and fungal communities were strongly different in their composition among discolored wood samples (similarities ranged from 16–46% and 1–62%, respectively) (Figure 2c,d). An exception was detected for fungal communities between samples D1 and D3, which showed 60% similarity.

Prokaryotes were dominated by bacteria, which accounted for 99% (627 out of 632) of total detected prokaryotic ASVs (Table S2). Four bacterial phyla were the most detected: Proteobacteria (212 ASVs), Firmicutes (146 ASVs), Bacteroidota (120 ASVs), and Actinobacteriota (78 ASVs). Five archaeal ASVs belonged to *Methanobacterium* spp. (2 ASVs), and *Methanomassiliicoccus* sp. (1 ASV), which were detected only from one discolored wood sample (D4) with relative abundances of 1–2.6%, whereas Nitrososphaeraceae (2 ASVs) were detected in two non-discolored wood samples (ND3 and ND4) with very low relative abundances (0.02–0.1%). Noteworthy, the anaerobic methanogenic archaea were only detected in the discolored wood while the ammonium oxidizing aerobic archaea were associated with non-discolored sapwood. Fungi were dominated by Ascomycota (69 out of total 124 fungal ASVs) and Basidiomycota (53 ASVs) (Table S1).

**Figure 3:**
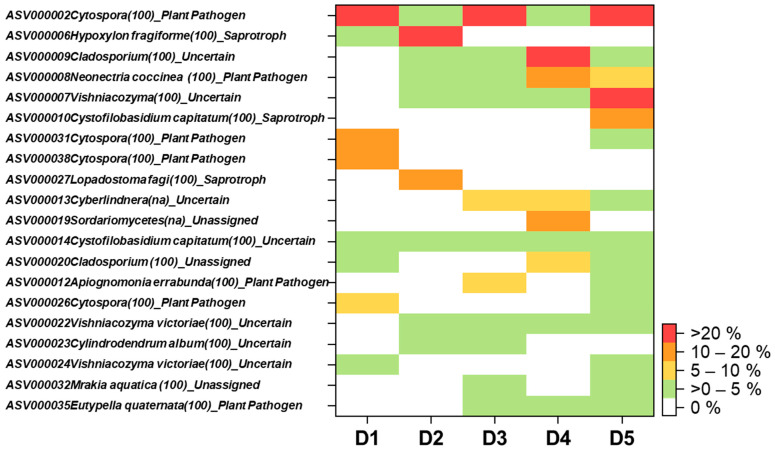
Heatmap of top 20 fungi with highest relative abundances detected in discolored wood areas (D) of European beech stumps located in Thüringen forest. The number in the parentheses after the name of each fungus indicated the percent identity matches with the current version of UNITE (version: 8.2). The UNITE species hypotheses are presented in Supplementary Table S3. Identification at species level was based on (i) 100% identity matches with the current version of UNITE; (ii) UNITE species hypotheses; and (iii) geographical locations.

All together, we detected 16 plant-pathogenic fungal ASVs, which contributed 2.2–97.6% of the total detected fungal sequences across five discolored wood samples (Table S1). Although similar symptoms have been observed for all selected beech trees, highly detected plant pathogenic fungi differed among different samples. *Cytospora* spp. were dominated in 3 out of 5 samples (D1 (97.6% of total detected fungal sequences), D3 (71.6%) and D5 (23.5%)) (Figure 3). *Neonectria coccinea* was detected in 4 out of 5 samples, but only dominated the sample D4 (16.8%) and co-dominated (9%) with *Cytospora* spp. in sample D5 (Figure 3). Sample D2 highly dominated by a saprotroph fungus *Hypoxylon fragiforme* (relative abundance = 80%) with little contribution of *Cytospora* sp (1.8%) and *Neonectria coccinea* (0.4%) (Figure 3). These results suggest that *Neonectria coccinea* may partly contribute to the beech decline in this Thüringen forest. In a previous study, *Neonectria coccinea* has been considered a common and widespread species in the northern hemisphere, frequently detected in the wood of beech and other deciduous trees, dung, and soils [37,38]. *Neonectria coccinea* is one of the most serious pathogens of beech trees, causing beech bark disease in North America and Europe [37,39]. A recent pathogenicity test also showed that *Neonectria coccinea* has a high potential to cause necrosis in European beech and may lead to tree mortality [40]. Furthermore, it has been hypothesized that damage caused by *Neonectria coccinea* will be increased under global warming scenarios, as many strains of this species show a rapid increase of mycelial growth at relatively high temperature (30 °C) [37,40]. We did not detect any *Phytophthora* spp. in our Illumina sequencing data, although the fungal primer pair used in this study (fITS7 and ITS4) has been reported to amplify *Phytophthora* spp. in environmental samples [41]. *Phytophthora* spp. have been reported to associate with decline of European beech, as well as other broadleaved trees in Europe occurring after drought events [42,43]. Thus, we also used *Phytophthora* genus and species specific primers to validate the existence/absence of *Phytophthora* spp. in wood samples [35]. The species specific primers included several major species of *Phytophthora* spp. (including, *Phytophthora cambivora*, *Phytophthora cactorum* and *Phytophthora citricola*) reported previously to cause beech decline in Europe [42,44]. The absence of *Phytophthora* spp. in our wood samples was confirmed by PCR with specific primers.

In this study, we identify *Cytospora* spp. as new candidates, which may also contribute to beech decline in this Thüringen forest. *Cytospora* spp. are important broad host range pathogens commonly associated with tree dieback and canker disease detected worldwide [45]. Furthermore, *Cytospora* spp. are known to positively respond to drought [46]. Nevertheless, the genus *Cytospora* includes organisms that are not strict pathogens, but can change their trophic-mode to saprotroph [45].

*Hypoxylon* is an additional genus that was identified in discolored wood and it is generally considered either endophyte or saprotroph, but some species (i.e., *Hypoxylon fragiforme*) can be associated to the development of necrotic lesions on wood trunk of European beech [40]. *Hypoxylon fragiforme* is even classified as a latent invader within the xylem of European beech [47]. *Hypoxylon fragiforme* is reported to co-occur with *Neonectria coccinea* [40]. In our study, we observed strong enrichment of *Hypoxylon fragiforme* only in discolored wood areas of sample D2 and it was almost absence (detected 8 times) in non-discolored wood areas of the same beech tree (ND2). We also detected *Neonectria coccinea* in the sample D2 which confirms that *Hypoxylon fragiforme* has the potential to co-occur with *Neonectria coccinea* [40].

**Figure 4:**
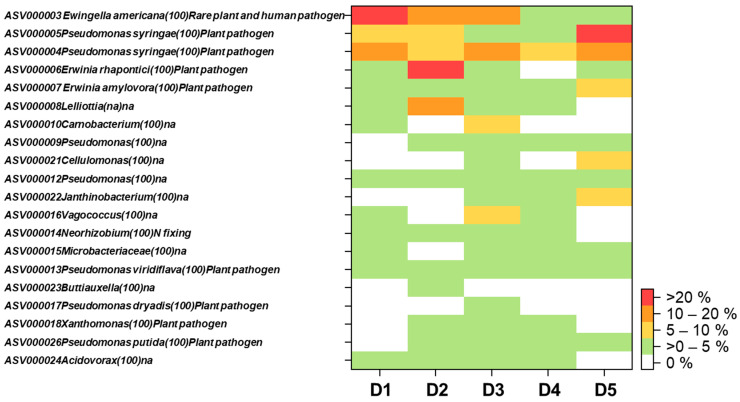
Heatmap of top 20 bacteria with highest relative abundances detected in discolored wood areas (N) of European beech stumps located in Thüringen forest. The number in the parentheses after the name of each bacterium indicated the percent identity matches with the current version of NCBI (version: 2.11.0).

In addition to unravelling the plant pathogenic fungi associated with beech decline, we frequently detected bacterial genera (i.e., *Acidovorax*, *Erwinia*, *Pseudomonas*, *Xanthomonas*) (Figure 4, Table S2) known to be potential pathogens in discolored wood samples [48]. In fact, 11 out of 20 most abundant bacterial ASVs detected in this study belonged to such potential plant pathogenic bacterial genera (average relative abundances = 44.6%, ranged from 16.3–76.9% in all five discolored samples) (Figure 4). *Erwinia* spp. and *Pseudomonas* spp. were detected in all discolored wood samples. Specifically, *Pseudomonas* ASV000004 and ASV000005 (closest hit (100%) with *Pseudomonas syringae*) co-dominated or highly dominated the bacterial communities in all discolored wood samples (relative abundances ranged 11.4–60.6%) (Figure 4). *Erwinia* ASV000006 and ASV000007 (closest hit (100%) with *Erwinia rhapontici* and *Erwinia amylovora*, respectively) were highly dominating in sample D2 (43.3% of total detected bacterial sequences) and substantially contributed in other 3 samples (D1, D3 and D5 with relative abundances ranged from 4.2–8.3%) (Figure 4). *Pseudomonas* spp., especially various pathovars of *Pseudomonas syringae* are identified as main disease agents of bacteria blight, canker and dieback for woody plants in agroforest ecosystems [49]. *Erwinia* spp. are important plant pathogenic bacteria associated with fire blight disease in woody plants, especially rosaceous hosts [50]. It has been shown that *Erwinia amilovora* (the most intensively studied species in this genus) can enter plant tissues via blossoms and stomata or wounds to above- or below-ground organs, and later it can spread systemically through the vascular system [51]. Moreover, from below ground *Erwinia amylovora* can also form aggregates/biofilms on tree root surfaces, colonize and infect the internal tissues of the roots, and later it can enter the stem tissues by xylem penetration [52]. Overall, our results suggest that plant pathogenic bacteria can potentially be an additional agent for beech decline, especially for weak beech trees suffering from drought, which may already have wounds from fungal infection or insect infestation. The bacterial agents for beech decline may have been overlooked as fungal agents are also present at the same infection areas.

Based on the results from our study, we hypothesize that fungal pathogens (i.e., *Cytospora* sp, *Neonectria coccinea*) may open the wounds of beech trees and bacterial pathogens get in through these wounds and colonize the base of the stem. Furthermore, the base of the stem is often affected by forest management practices (i.e., wounds caused by physical injury), thus bacterial pathogens can also get in through such wounds. Interactions between insects and plant pathogens should also receive more attention as they have been reported to strengthen the severity of diseases and cause higher mortality rates to beech trees [42,44,53]. Specifically, wounds (especially, those produced by insects) open the route to infection of woody organs by fungal species that are not able to directly penetrate undamaged tissues [42].

In our study, we analyzed the pathogens in red to dark brown discolored wood areas located in the tree stump, as we have observed such symptom consistently across all dead beech trees. However, possible causal agents of beech decline may still remain unexplored. For example, some *Phytophthora* spp. can kill lateral roots and fine roots [42], and cause beech decline without invading xylem. Furthermore, some other plant pathogenic fungi belonging to Diaporthales and Botryosphaeriales can kill twigs and branches and cause crown-dieback [23,40,54]; thus, they may not be detected in stump samples. Due to this reason, future studies should focus on all potential routes of pathogens from above (stems, branches, twigs and leaves) and below ground organs of the trees and including surrounding soils and insects. This will enable us to draw a correct conclusion on the causal pathogens of beech decline and how these bacterial and fungal plant pathogens enter beech trees. Pathogenicity and aggressiveness of beech decline pathogens should be quantified by inoculation tests using pathogens isolated from all around the trees under drought conditions. Studying the whole tree is difficult because the damage is not visible from the outside, and emerges only when cutting the tree. However, sampling at this point is very dangerous due to wood cutting operations.

The detected presence of fungal and bacterial pathogens across all samples confirms that Illumina sequencing is a very sensitive method to detect plant pathogenic organisms in environmental samples. The detection limit of the Illumina MiSeq System currently reaches approximately 10 microbial cells per mL [55]. Nevertheless, keeping the possible biases of short-read NGS in mind, our results provide only a quick snapshot on potential plant pathogenic fungi and bacteria (taxonomic information valid at least at the genus level) associated with beech decline in a forest of Thuringia. Future studies should combine both culture-dependent techniques and culture-independent molecular techniques (i.e., NGS or third-generation sequencing) to investigate the tree decline at a larger scale and to fulfil Koch’s postulates. Interactions between plant pathogenic fungi and bacteria co-occurring in beech, as well as other forest tree species under drought conditions, are worth investigating.

## 4. Conclusions

In conclusion, our work demonstrates that European beech decline after an extreme drought event in 2018 and sampling in 2019 is associated with both bacterial and fungal plant pathogens. In addition to pathogenic fungi, red to dark brown discoloration areas emerge as hotspot for plant pathogenic bacteria. However, almost each tree was individually associated with a different combination of pathogens. *Cytospora* spp. and *Neonectria coccinea* were among the most frequently detected fungal pathogens, whereas *Erwinia* spp. and *Pseudomonas* spp. were the dominant plant pathogenic bacteria. We currently do not know how these plant pathogenic fungi and bacteria interact, but all beech trees that contain high abundances of these pathogens eventually died within a year of the extreme drought event in 2018. The absence of *Phytophthora* spp. in our study may be due to the specific environmental conditions of our study area and interactions between *Phytophthora* spp. and other local wood-inhabiting fungi and bacteria.

## Data Availability

The Illumina sequencing of prokaryotic and fungal datasets are deposited in The National Center for Biotechnology Information (NCBI) database under BioProject ID: PRJNA722031.

## Supplementary Materials

The following are available online at https://www.mdpi.com/article/10.3390/plants10102092/s1, Figure S1: Wood samples used in this study and characteristics of discolored and non-discolored wood samples. Figure S2: Rarefaction curves of non-discolored (ND) samples, Table S1. Taxonomic and abundance (sequence reads) of all fungal ASV detected in discolored and non- discolored beech wood samples, Table S2. Taxonomic and abundance (sequence reads) of all bacterial ASV detected in discolored and non- discolored beech wood samples. Table S3. UNITE species hypotheses of the top 20 fungal ASVs with highest relative abundances detected in discolored wood areas (D) of European beech stumps located in Thüringen forest.
