# Supplementary material for: Molecular Screening of Microorganisms Associated with Discolored Wood in Dead European Beech Trees Suffered from Extreme Drought Event Using Next Generation Sequencing
Source: Plants (Basel). 2021 Oct 2;10(10):2092. doi: 10.3390/plants10102092 (PMC8537330; doi:10.3390/plants10102092)
Supplement: Supplementary file 1 [file plants-10-02092-s001.zip › plants-1335629-supplementary/Supplementary Information_rev.pdf]

Supplementary Information

Molecular screening of microorganisms associated with discolored wood in dead European beech trees suffered from extreme drought event using Next Generation Sequencing

Witoon Purahong[1]*, BenjawanTanunchai[1], Sara Fareed Mohamed Wahdan[1, 2], François Buscot[1,3], Ernst-Detlef Schulze[4]

Affiliations:

[1]UFZ-Helmholtz Centre for Environmental Research, Department of Soil Ecology, Theodor-Lieser-Str. 4, D-06120 Halle (Saale), Germany

[2]Botany Department, Faculty of Science, Suez Canal University, 41522 Ismailia, Egypt

[3]German Centre for Integrative Biodiversity Research (iDiv) Halle ‐ Jena ‐ Leipzig, Deutscher Platz 5e, D ‐ 04103 Leipzig, Germany

[4]Max Planck Institute for Biogeochemistry, Hans-Knöll-Str. 10, D-07745 Jena, Germany

*Correspondence and requests for materials should be addressed to Witoon Purahong (Tel: 0345 558 5207, e-mail: witoon.purahong@ufz.de).

Fig. S1 Wood samples used in this study and characteristics of discolored and non-discolored wood samples. Wood samples were frozen at -80 °C, thus colour of discolored wood appears more subtle and less intense as compared with fresh samples.

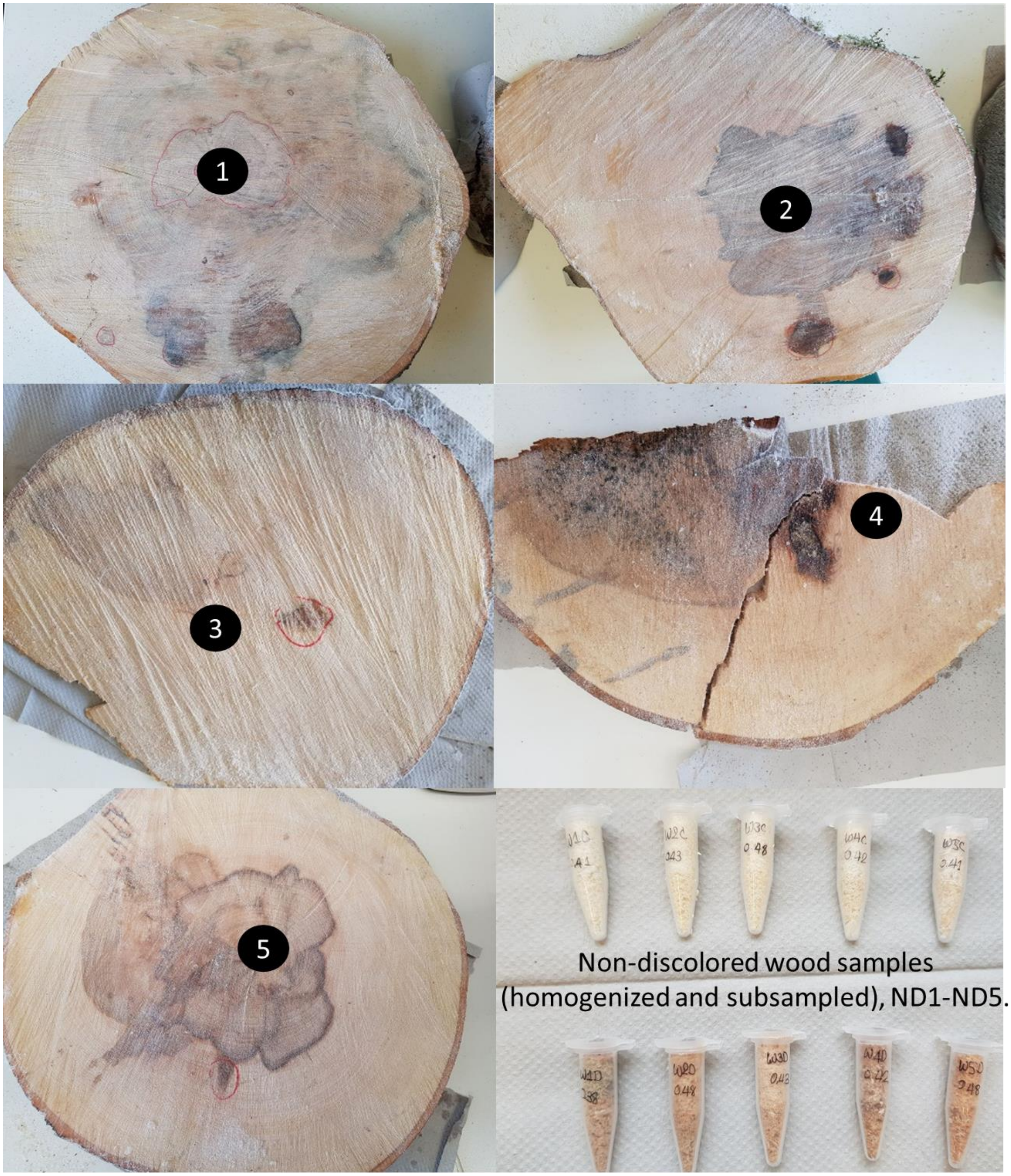

Fig. S2 Rarefaction curves of non-discolored (ND) wood samples.

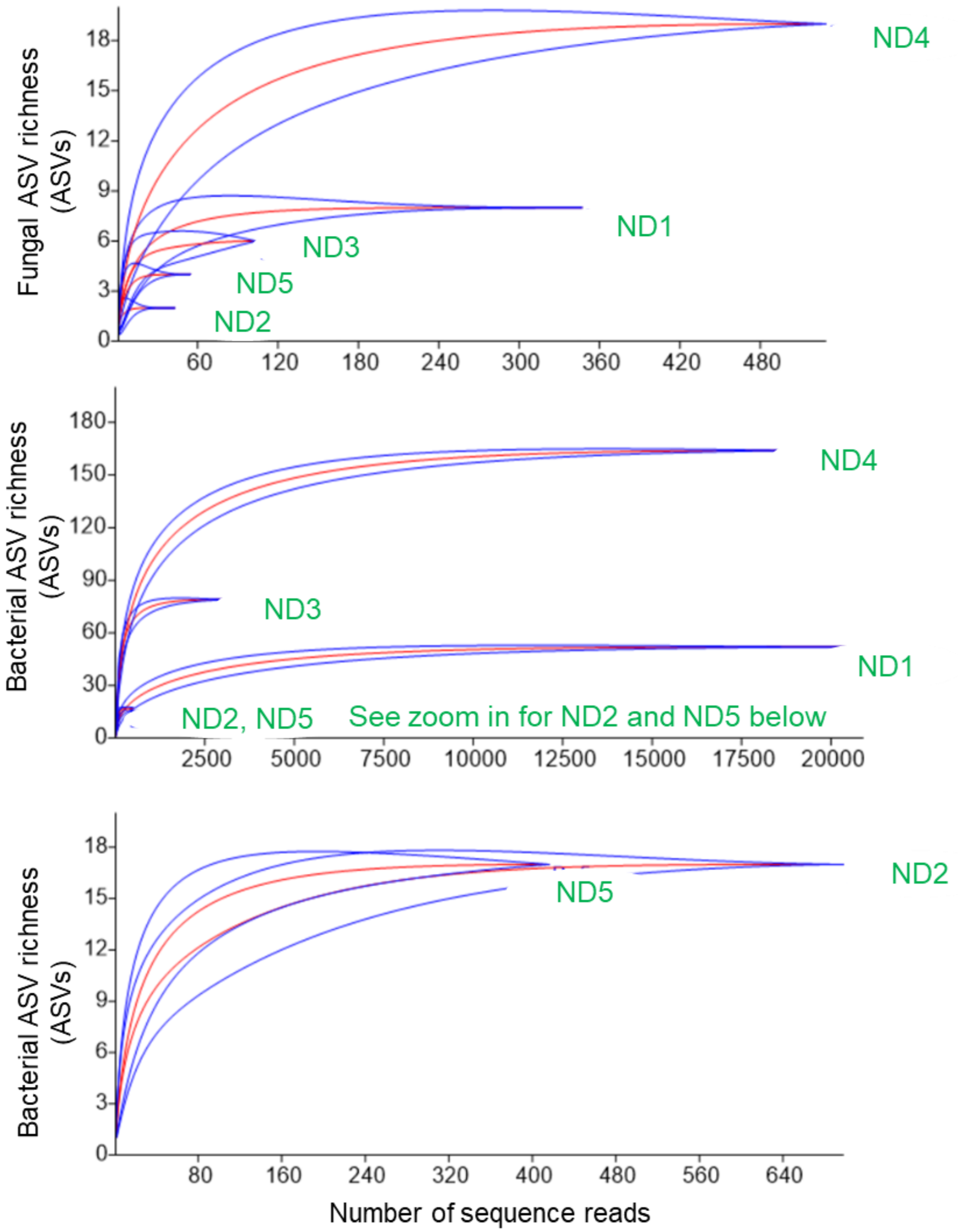

Table S1. Taxonomic and abundance (sequence reads) of all fungal ASV detected in discolored and non-discolored beech wood samples.

Table S2. Taxonomic and abundance (sequence reads) of all bacterial ASV detected in discolored and non-discolored beech wood samples.

Table S3. UNITE species hypotheses of the top 20 fungal ASVs with highest relative abundances detected in discolored wood areas (D) of European beech stumps located in Thüringen forest.
